# IL-1β Induced Intestinal Inflammation Pathogenesis in East Friesian Sheep: Insights from Organoid Modeling

**DOI:** 10.3390/ani15081097

**Published:** 2025-04-10

**Authors:** Yue Xue, Yulong Zhao, Shuo Yan, Ruilin Du, Huiming Zhang, Wenna Yao, Teligun Bao, Fei Pan, Siqin Bao, Xihe Li, Yongli Song

**Affiliations:** 1 Research Center for Animal Genetic Resources of Mongolia Plateau, College of Life Sciences, Inner Mongolia University, Hohhot 010020, China; 14794835014@163.com (Y.X.); z1369609005@outlook.com (Y.Z.); yanshuo202305@163.com (S.Y.); rallingd@163.com (R.D.); zhanghuimin202404@163.com (H.Z.); yaowenna024@163.com (W.Y.); 13947356727@163.com (T.B.); pf930829@163.com (F.P.); baosq@imu.edu.cn (S.B.); 2 The State Key Laboratory of Reproductive Regulation and Breeding of Grassland Livestock, College of Life Sciences, Inner Mongolia University, Hohhot 010020, China; 3 National Center of Technology Innovation for Dairy Industry, Hohhot 010020, China; 4 Inner Mongolia Saikexing Institute of Breeding and Reproductive Biotechnology in Domestic Animal, Hohhot 011517, China

**Keywords:** IL-1β, intestinal inflammation organoid, intestinal stem cells, RNA-seq, NF-Κappa B/TNF/IL-17 signaling pathway

## Abstract

Establishing an appropriate model of intestinal inflammation in vitro is crucial for studying the pathogenesis of inflammatory bowel disease (IBD). While immortalized cell lines have long been employed to investigate intestinal cell functions and host-pathogen interactions in sheep, they inadequately mimic the physiological characteristics of ovine intestinal inflammation and may lose critical genetic features. The emergence of organoids has revolutionized in vitro intestinal inflammation modeling by offering superior structural and functional fidelity to native tissues. In this study, we developed an IL-1β-induced intestinal inflammation organoid model derived from East Friesian sheep ileal crypts. Our data demonstrate that treatment with 30 ng/mL IL-1β significantly increased inflammatory factor secretion, the apoptosis of intermediate cells, and disrupted barrier integrity while simultaneously reducing bud formation capacity, organoid area, and stem cell proliferation. RNA-seq analysis revealed that IL-1β activated the NF-κB/TNF/IL-17 signaling pathway in intestinal organoids, thereby orchestrating the inflammatory response. This study establishes a novel sheep-derived intestinal inflammation organoid model, providing a physiologically relevant platform to investigate intestinal inflammation pathogenesis. These findings offer a translational tool for advancing drug development and pharmacological mechanism exploration in ovine intestinal inflammation research.

## Simple Summary

Establishing appropriate models of intestinal inflammation in vitro is essential to studying the pathogenesis of inflammatory bowel disease (IBD). Organoids have revolutionized gut inflammation modeling by providing superior structural and functional fidelity to native tissues. Here, we developed an IL-1β-induced intestinal inflammation organoid model derived from the ileal crypt of East Friesian sheep. RNA-seq analysis revealed that IL-1β activated the NF-κB/TNF/IL-17 signaling pathway, thereby coordinating the inflammatory response. In this study, a novel sheep-derived organoid model of intestinal inflammation was established, which provides a physiologically relevant platform for studying the pathogenesis of intestinal inflammation. These findings provide translational tools to advance drug development and pharmacological mechanism exploration in sheep intestinal inflammation research.

## 1. Introduction

Inflammatory bowel disease (IBD) is a group of intestinal diseases characterized by chronic inflammation, posing severe threats to the health and survival of large animals. In human or animal intestinal tissues, various cell types, including epithelial cells and immune cells, work together to maintain the integrity of the intestinal barrier and regulate immune homeostasis. However, during IBD pathogenesis, cellular composition becomes imbalanced, functional impairment occurs, and intercellular interactions are disrupted, leading to intestinal barrier breakdown and immune dysregulation, thereby exacerbating inflammation. The etiology of IBD remains unclear, with complex progression mechanisms influenced by genetic, immunological, and microbial factors [1,2]. Current treatments primarily target inflammation control, including amino acid derivatives, immunomodulators, glucocorticoids, biologics, small-molecule drugs, and traditional Chinese medicine. While these therapies show efficacy, individual patient responses vary significantly due to IBD’s heterogeneous nature, and some patients exhibit drug tolerance. Thus, there is an urgent need to develop novel therapeutics or strategies based on deeper pathological insights.

Animal models have historically served as primary tools for studying IBD mechanisms and drug evaluation, offering holistic biological insights. However, limitations such as low humanization, inter-species physiological differences, and high costs restrict their application in large-scale drug screening [3]. In contrast, cell-based in vitro models provide direct pathological insights into IBD with advantages including high efficiency, cost-effectiveness, and scalability [4]. Recent advances in cell culture and tissue engineering have enabled the development of diverse IBD models, such as immortalized cell lines, co-culture systems, organoids, and organoid chips. These models better recapitulate in vivo intestinal pathology and facilitate drug discovery and mechanism exploration [5,6].

Intestinal organoids, also known as mini-gut, are hollow cell clumps formed via the proliferation and differentiation of intestinal stem cells or crypts in the matrix gel containing special growth factors with similar structure and function to the intestine. In intestine organs, huge regenerative capacity is mediated by the proliferation and differentiation of tissue resident adult stem cells (ASCs) [7,8,9,10]. In intestinal tissues, They mimic intestinal architecture and function, with adult stem cells (ASCs) residing in crypt bases expressing leucine-rich repeat-containing G protein-coupled receptor 5 (LGR5). These ASCs differentiate into five epithelial lineages: enterocytes, goblet cells, enteroendocrine cells, tuft cells, and Paneth cells [8,9].

Organoids are 3D miniaturized representations of selected tissues in a petri dish [11]. A three-dimensional (3D) cell culture system seems to be more promising because it has a higher level of cell differentiation and tissue organization, very similar to tissues and organisms [12]; organoids represent a 3D microenvironment where cells are embedded in an extracellular matrix (ECM), supporting tissue-specific signaling and homeostasis [13]. ECM supports a tissue-specific microenvironment, which is essential for cell differentiation and tissue maintenance [14]. This system has revolutionized tissue engineering and drug testing across various organs [15].

Bovine small intestinal crypts can inform organoids in a 3D culture medium [16]. We, therefore, established East Friesian sheep ileal epithelial organoids representing critical intestinal sites for infection. Our data demonstrate stable self-organization of ileal organoids with a single epithelial layer and central lumen over multiple passages. RNA-seq analysis revealed transcriptomic profiles of these organoids, and we validated long-term cryopreservation and storage protocols, reducing animal dependency.

Proinflammatory cytokines like interleukin-1β (IL-1β) and tumor necrosis factor-α (TNF-α) are markedly elevated in IBD [17]. IL-1β is crucial for host-defense responses to infection and injury [18]. IL-1β, produced primarily by innate immune cells (e.g., macrophages), exists as a 31-kDa pro-form activated via PAMP/DAMP signaling [19]. Although its priming and secretion pathways are partially understood, the exact mechanism remains unclear. We aim to establish an IL-1β-induced inflammation model using East Friesian sheep intestinal organoids to elucidate pathogenic mechanisms.

Research shows that researchers employed a well-established in vitro intestinal epithelial model system, namely, Caco-2 intestinal epithelial monolayers cultured on filters. They discovered that IL-1β within the concentration range of 0 to 100 ng/mL led to a concentration and time dependent decrease in the transepithelial electrical resistance of Caco-2 cells. Notably, 30 ng/mL of IL-1β, falling within the concentration range where significant effects could be observed, caused a gradual increase in the transepithelial permeability of paracellular markers over time. This indicates the induction of a robust inflammatory response [20]. This study provides an IL-1β concentration range for our experiment design.

## 2. Materials and Methods

### 2.1. Animals

All animal procedures were conducted in accordance with the institutional guidelines and regulations for animal care and use. The study was approved by the Institutional Animal Care and Use Committee of Inner Mongolia University. The approval number is NMGDX 2022-0003. The ileum tissue of East Friesian sheep was derived from a 1-month-old Homozygous male lamb.

### 2.2. Isolation of East Friesian Sheep Intestinal Crypts

Tissues were removed from East Friesian sheep by surgery. Ileal tissue sections, with an approximate length of 10 cm, were retrieved from a site positioned approximately 30 cm in the distal direction relative to the ileocecal junction. Tissues were placed into sterile ice-cold Dulbecco’s Phosphate-Buffered Saline (DPBS) containing 2.7% gentamicin (G1272-10 ML; Sigma-Aldrich, St. Louis, MO, USA) and 1% penicillin/streptomycin (15140122; Gibco, Grand Island, USA), an antibiotic mix was applied to inhibit bacterial growth (hereinafter referred to as Gen-2.7% and P/S-1%). Using a sterile scalpel and forceps to expose the epithelial surfaces, dissection scissors were employed to perform a longitudinal incision on the ileum. Rinsing the luminal surfaces with DPBS was carried out to get rid of digesta, following which they were placed on sterile Petri dishes [21]. The majority of the mucus layer was delicately detached using a glass slide. Subsequently, the surface mucosal tissue, which encompasses intestinal crypts, was gathered by firmly scraping with a new glass slide [21]. Following this, the mucosal tissue was transferred into a Falcon tube containing 50 mL of DPBS supplemented with Gen-2.7% and P/S-1%. Samples underwent centrifugation at 300× *g* for a duration of 3 min. This centrifugation process yielded a tissue pellet with a mucus layer positioned on its upper surface. Subsequently, the supernatant, along with the mucus layer that had formed on top, was carefully aspirated and discarded. The tissue was then resuspended in 50 mL of DPBS, which was pre-supplemented with Gen-2.7% and P/S-1%. The procedures of centrifugation, aspiration, and resuspension were carried out iteratively. This continued until no mucus layer could be observed over the tissue pellet [21]. To liberate intestinal crypts from the tissue, the tissue pellets were resuspended in 25 mL of the digestion medium. The digestion medium consisted of DMEM/F12 (11320-033; Gibco, Grand Island, USA), 1% BSA, 4 mM EDTA (E5513; Sigma, Saint Louis, USA), Gen-2.7%, and P/S-1%. Subsequently, the suspension was incubated horizontally in a shaking incubator at 37 °C with a rotation speed of 80 revolutions per minute (rpm) for 30 min. After the digestion process, the tube was gently agitated to detach the cells and then left at room temperature for a short period to enable the large tissue debris to sediment.

The supernatant was carefully transferred into a sterile 50 mL Falcon tube. Subsequently, the integrity of the glands and crypts present in the supernatant was evaluated using light microscopy. Samples were then centrifuged at 300× *g* for 3 min, with the resulting supernatant containing released crypts. The crypt-containing supernatant was washed by centrifugation at 300× *g* for 3 min, and the crypts were resuspended in 1–2 mL advanced DMEM/F12 (12634-010; Gibco, Grand Island, USA) containing 0.1%BSA, Gen-2.7% and P/S-1%.

### 2.3. Organoid Culture

A quantity ranging from 200 to 1000 intestinal crypts was resuspended in 100 µL of DMEM/F12 medium. This medium was formulated with 0.1% bovine serum albumin (BSA), Gen-2.7%, and P/S-1%. Subsequently, this suspension was added to 230 µL of Growth Factor Reduced Matrigel Matrix (082701; Abwbio, Shanghai, China). Fifty-microliter droplets were dispensed into sequential wells of a 24-well tissue culture plate (Corning, New York, NY, USA). Plates were incubated at 37 °C, 5% CO_2_ for 30 min to allow the Matrigel to polymerize and then 500 µL of prewarmed complete IntestiCult Growth Medium (mouse) (6005; STEMCELL Technologies, Vancouver, BC, Canada) containing 10 µM Y-27632 (HY-10071; MCE, Shanghai, China), 10 µM LY2157299 (SF7926; Beyotime, Shanghai, China) [21], 25 µg/mL FGF-basic (Z03230; GenScript, Nanjing, China), 100 ng/mL IGF-1 (Z03688; GenScript, Nanjing, China), Gen-2.7%, and P/S-1% added to each well. The plates were subjected to incubation at 37 °C within an environment of 5% CO_2_ to facilitate the development of organoids. The complete IntestiCult medium was renewed every 2 to 3 days. Generally, organoids were cultivated for a period ranging from 7 to 10 days before passage. Throughout the 9-day in-vitro growth phase, phase-contrast microscopy was employed to capture images of the organoids.

### 2.4. Organoid Passage

The IntestiCult medium was removed from the cultured organoids, and the Matrigel matrix was dissolved by replacing it with 1 mL DPBS. The resuspended organoids were transferred into a 15 mL centrifuge tube, and the total volume of DPBS was increased to 10 mL. The samples were placed on ice for 5 min to allow the organoids to sediment, after which the supernatant was removed. The organoids were resuspended in 200 μL of DPBS containing Gen-2.7% and P/S-1%. Mechanical disruption was then carried out by repeatedly pipetting (approximately 30 times) using a 200 μL pipette tip. The number of organoid fragments was counted via light microscopy, and the samples were diluted to a concentration of 200–1000 crypts per 100 μL. Subsequently, 100 μL of the liquid was combined with Matrigel and seeded into 24 well or 48 well tissue culture plates. Microscopy was utilized to image the organoids from passage 1 to passage 5, with each passage undergoing 7 days of in vitro growth.

### 2.5. Organoid Cryopreservation

The IntestiCult media in the cultured organoid system was evacuated, and the Matrigel matrix was dissolved by substituting it with 1 mL of ice-cold DMEM/F12 medium. The organoids in resuspension were conveyed to a microcentrifuge tube and centrifuged at 300× *g* for a 3 min duration at 4 °C, resulting in the formation of a pellet. After the completion of the centrifugation step, the supernatant was carefully aspirated away. The organoid pellets were then resuspended in Cebrary^®^Cell freezing medium for Organoid (41422ES60, TEASEN, Shanghai, China) at a concentration of roughly 500–1000 organoids per milliliter. Subsequently, this suspension was transferred into a cryovial. The cryovials were placed within a cryogenic freezing container and maintained at −80 °C for a duration of 2 h. Thereafter, they were relocated to −196 °C for extended-term preservation.

Cryopreserved organoids underwent resuscitation through the process of thawing the cryovials in a 37° C water bath. Subsequently, the organoids were swiftly transferred into a 15 mL Falcon tube that held 8 mL of DMEM/F12 medium (Gen-2.7% and P/S-1%). An additional 1 mL of the medium was used to rinse the cryovial, and the resulting liquid was incorporated into the Falcon tube. The samples were then subjected to centrifugation at 300× *g* for 5 min at 4 °C to form a pellet. Subsequently, the pellet was resuspended in 200 µL of freshly prepared DMEM/F12 medium (containing Gen-2.7% and P/S-1%). Resuspended organoids were added to the Matrigel and cultivated. Prior to cryopreservation and subsequent to it, the organoids were visualized using phase-contrast microscopy after undergoing 7-day in vitro growth.

### 2.6. Screening of IL-1β Treatment Concentration

To establish an inflammatory model of ileum organoids in East Friesian sheep, we treated the organoids with different concentrations of IL-1β (0 ng/mL, 10 ng/mL, 20 ng/mL, 30 ng/mL, and 40 ng/mL). By systematically evaluating the growth status, bud-ding rate, degree of differentiation, and apoptosis level of the organoids, we aimed to screen out the optimal concentration that could induce a moderate inflammatory response (Supplementary Figure S1). The experimental results showed that with the increase of IL-1β concentration, the activity of organoids decreased significantly in a dose-dependent manner. At concentrations of 10 ng/mL and 20 ng/mL, although the growth and budding ability of organoids were weakened, the apoptosis was not significant, and the purpose of simulating the inflammatory state was not achieved. However, at a concentration of 30 ng/mL, the organoids showed moderate apoptotic characteristics while still maintaining certain activity and structural integrity, which could better simulate the intestinal tissue response under inflammatory conditions. In contrast, in the 40 ng/mL treatment group, the activity of organoids was significantly reduced, and the growth and differentiation abilities were basically lost, indicating that this concentration caused too much damage to the organoids and was not suitable for subsequent experiments. Based on the above results, we finally selected 30 ng/mL IL-1β treatment for 24 h as the optimal inflammatory induction condition, and all relevant experiments in this paper were carried out at this concentration. (The concentration screening of IL-1β is shown in Figure S1 in the Supplementary Materials).

### 2.7. Total RNA Extraction

The IntestiCult medium was removed from the wells of mature cell cultures and replaced with 1 mL of DPBS. The resulting suspension containing the dissolved Matrigel and organoids was transferred to a 15 mL sterile centrifuge tube and brought up to 10 mL with ice-cold DMEM/F12. The organoids were pelleted by centrifugation at 300× *g* for 5 min, and the supernatant was removed. The organoid pellet was resuspended in 1 mL of DPBS containing 0.1% BSA, Gen-2.7%, and P/S-1%, followed by centrifugation at 12,000× *g* for 5 min. The supernatant was then removed, and the pellet was stored at −80 °C.

Total RNA was isolated from each sample using the RNeasy Mini Kit (74104, Qiagen, Hilden, Germany) according to the manufacturer’s protocol. On-column DNase digestion was selected, and the total RNA was eluted with 30 μL of nuclease-free water. The total RNA extracted in each case was quantified using a NanoDrop™ One spectrophotometer. The purified total RNA was stored at −80 °C, and subsequent experiments were carried out

### 2.8. RNA-Seq Analysis

For each sample, 1 μg of total RNA was used for RNA-seq analysis. All library construction and sequencing were carried out at Omicshare. A total of six libraries were constructed [including ovine ileum organoids P0–P4 (The control group and the treatment group were randomly grouped at one stage), *n* = 3].

### 2.9. Immunohistochemistry

Ileum organoids were cultivated in Matrigel for 7 days in 8-well chamber slides (Millipore). To render the organoids amenable to immunohistochemistry reagents, the existing culture medium was evacuated and substituted with ice-cold 4% paraformaldehyde. For the purpose of fixation, the samples were held at 4 °C for 20 min period. This not only dissolved the Matrigel but also precluded its re-solidification. The organoids underwent three washes with IF buffer (PBS containing 0.1% Tween 20). Subsequently, they were made permeable using 0.1% Triton X-100 in PBS at room temperature for 20 min intervals. The samples were then subjected to 3 washes with the IF buffer. After that, they were blocked for 30 min with 1% BSA in the IF buffer at room temperature. Next, the primary antibodies, which were diluted in the blocking solution, were added to the organoids, and the samples were left to incubate overnight at 4 °C. Primary antibodies used included polyclonal rabbit Ki67 (PA5-19462, Invitrogen, 1:1000 dilution, Waltham, MA, USA.), polyclonal rabbit LYZ (GB11345, Servicebio, 1:200 dilution, Wuhan, China), Chromogranin A Monoclonal antibody (60135-2, Proteintech, 1:200 dilution, Wuhan, China), anti-TNF rabbit polyclonal antibody (D221347, BBI, 1:200 dilution, Markham, ON, Canada), mouse a-villin (sc-58897, Santa Cruz Biotechnology, 1:200 dilution, Dallas, TX, USA), and monoclonal mouse anti-β-catenin (c7207, Sigma,1:100 dilution). On the following day, the samples were rinsed three times with the IF buffer. Subsequently, the secondary antibodies, which were diluted at a ratio of 1:500 in the blocking buffer, were introduced, and the samples were incubated at room temperature for an hour. Secondary antibodies used were goat α-mouse Alexa Fluor 488 (ab150117, Abcam, Cambridge, UK) and goat a-rabbit Alexa Fluor 488 (ab150081, Abcam, Cambridge, UK).

The samples underwent three washes with the IF buffer, after which the DAPI solution was added. Nuclear Labeling solution is then added to label the nuclei (C02-04002, Bioss, Woburn, MA, USA). Prior to being washed 3 times with the IF buffer, the samples were incubated at room temperature for an additional 5 min. Finally, the mounting of slides was carried out by means of an Antifade Mounting Medium with DAPI (H-1200, VECTASHIELD, Burlingame, CA, USA) and imaged by confocal microscopy using a Nikon Ax upright laser confocal microscope (SMZ7457, Nikon, Tokyo, Japan) and the Nikon operating software(NIS Elements Viewer, Version 5.21.00).

## 3. Results

### 3.1. East Friesian Sheep Enteroids Culture

Ileal crypts were isolated from the ileum of healthy East Friesian sheep (≧1 month old), embedded in Matrigel, and cultivated in an Intesticult medium containing rho-related kinase inhibitor (Y27632), fibroblast growth factor (FGF), insulin-like growth factor (IGF-1), and TGF-βR inhibitor (LY2157299) in which the ileal crypts similarly closed and formed intestinal globs within 24 h, and by day 4 many crypt bud-like structures had formed around the central lumen [22]. IGF-1 is available at a high concentration in the human bloodstream [23] and has been shown to promote crypt expansion in mice [24]. FGF-2 is expressed in mesenchymal cells adjacent to intestinal crypts and is upregulated following tissue injury to promote regeneration [25]. These observations support IGF, FGF (IF), and their signaling pathways as in vivo-relevant niche factors for intestinal stem cells that enable the long-term culture of a range of intestinal stem cells.

**Figure 1:**
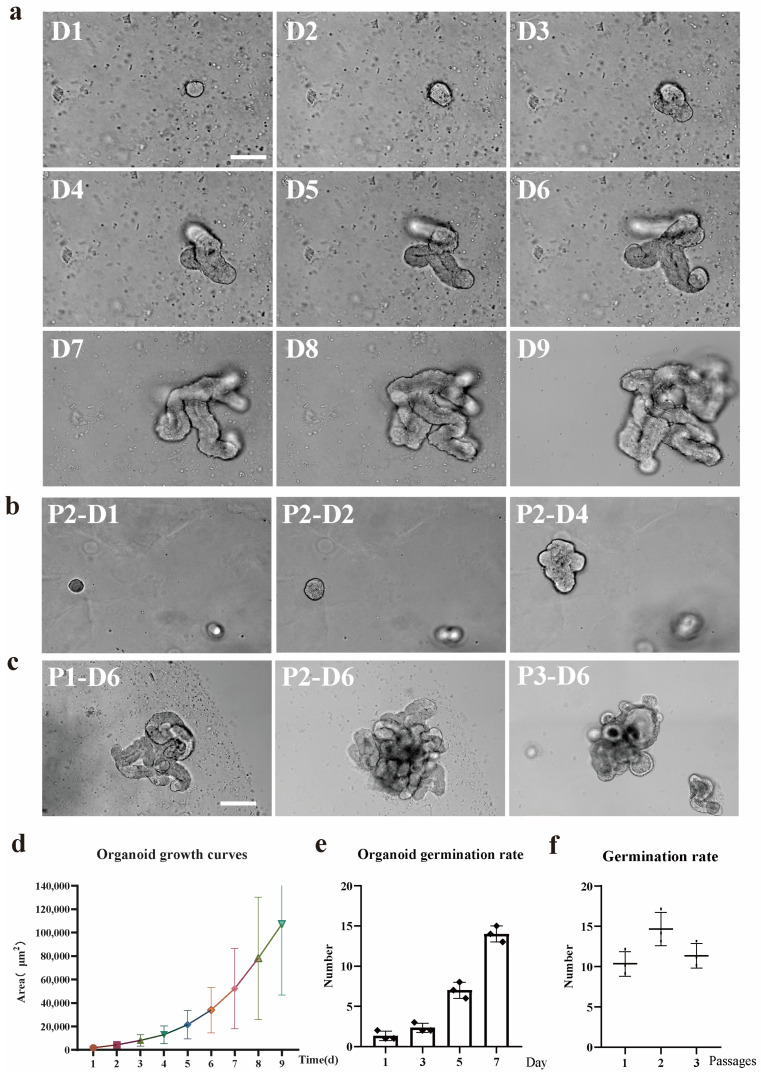
Sheep enteroids cultured in an Intesticult medium alone. (**a**) Representative images showing the growth and development of individual enteroids over 9 days of culture. Scale bar, 100 μm. (**b**) Second-generation growth state after passaging (P1: passage 1; D1: day 1 post-passage). Scale bar, 100 μm. (**c**) Growth status of organoids across days in the first three generations. (**d**) Statistical analysis of 9-day area changes in first-generation organoids (ANOVA, *p* < 0.05). (**e**) Statistical analysis of 7-day germination rates for first-generation organoids (ANOVA, *p* < 0.01). (**f**) Germination rate comparison among the first three generations (ANOVA, *p* < 0.05). Scale bar, 100 μm. (Concentrate: Figure 1d–f: Individual data plots are presented. Data are derived from three individual organoids distributed among three individual wells. These wells are from one different culture batch. A total of one animal was used in the experiment to generate the raw counts shown in the graphs.).

Within 24 h of culture, the upper opening of the crypts was sealed off. This suggested regional division and expansion of the crypt regions, and the formation of enteroid-like structures with a central lumen. Electron microscopy imaging revealed the size of the intestinal organoids gradually increased with culture duration (Figure 1a). After passaging, the organoids budded and grew normally (Figure 1b). Continuous passaging of the organoids was achieved (Figure 1c). During the 9-day area statistics of organoids, analysis using Image J software (Image J 1.54f )revealed that as time progressed, the area of organoids showed a significant growth trend. From day 1 to day 9, there were significant differences in the area of organoids at different time points (ANOVA, *p* < 0.05, GraphPad Prism 8.0.1), indicating that the culture time had a significant impact on the growth of organoids (Figure 1d). The germination rate of organoids was quantified over 7 days of continuous growth, showing an exponential increase with time elapsed (ANOVA, *p* < 0.01, GraphPad Prism 8.0.1); it more intuitively demonstrates that the increase in culture time significantly promotes the increase in the budding rate of organoids (Figure 1e). Comparative analysis of the germination rate across three consecutive generations revealed decreased germination from the third generation, although normal growth was sustained (ANOVA, *p* < 0.05, GraphPad Prism 8.0.1) (Figure 1f).

Many studies have demonstrated that enteroid cultures derived from mouse and human crypts can be long-term maintained through serial passaging. With extended culture, organoid size, and crypt bud frequency increased. Notably, enteroids could be mechanically dissociated and replated at a density of 1:3 every 7 days. Using this protocol, up to 5–8 generations of intestinal organoids were continuously propagated from individual donor lambs over 2 months, with no detectable changes in growth kinetics or morphology. Our data show that the cells were able to grow normally until the 8th passage. Due to time constraints, we have not conducted experiments beyond the 8th generation.

### 3.2. The Establishment of Intestinal Organoids Disease Models In Vitro

**Figure 2:**
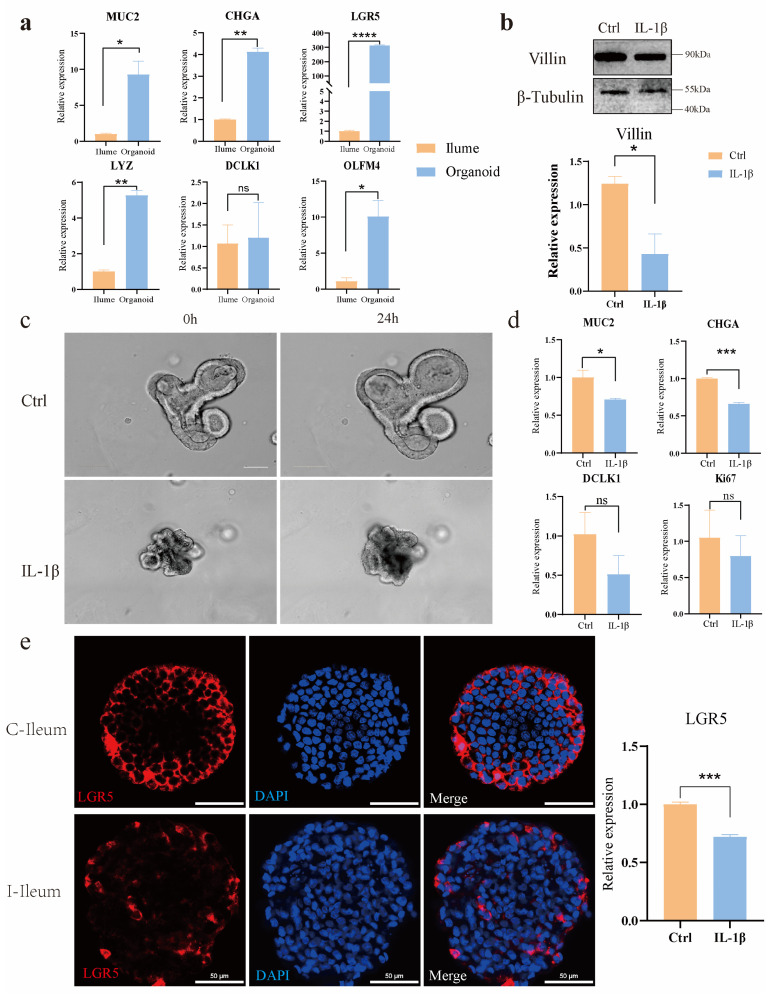
Comparative analysis of ileum tissue and ileum organoids from East Friesian sheep. (**a**) RNA was extracted from the ileum and ileal organoids of East Friesian sheep, and the differences in their expression levels were analyzed. (**b**) Changes in villin at the protein level and mRNA expression level. (**c**) Changes in the phenotype of organoids after treatment with 30 ng/mLIL-1β. Scale bar, 100 μm. (**d**) Changes in intestinal stem cell markers at mRNA levels, including MUC2, CHGA, DCLK, and Ki67. (**e**) Immunofluorescence staining and mRNA of intestinal stem cells LGR5. Scale bar, 50 μm. * *p* < 0.05, ** *p* < 0.01, *** *p* < 0.001, **** *p* < 0.0001.

In order to prove whether it is an organoid, we extracted RNA from the ileum and ileum organoids of East Friesian sheep and analyzed the differences in the expression of various intestinal epithelial cell markers, including goblet cells (MUC2), enteroendocrine cells (CHGA), intestinal stem cells (LGR5, DCLK1), etc., and the results showed that the intestinal epithelial cell markers were expressed in the organoids. Indicates that it is an intestinal organoid (Figure 2a). Villin, an epithelial-specific actin-binding protein, was detected in 7-day cultured ileal organoids, with reduced expression following IL-1β treatment, * *p* < 0.05 (Figure 2b). After 24 h 30 ng/mL IL-1β exposure, control organoids exhibited normal growth and differentiation, whereas IL-1β treated organoids showed increased apoptosis and growth arrest, validating the disease model’s phenotypic response (Figure 2c). qPCR analysis revealed a diminished expression of LGR5+ intestinal stem cells (ISCs), MUC2, CHGA, DCLK1 and reduced proliferation (Ki67), indicating IL-1β-induced inhibition of ISC self-renewal and differentiation, * *p* < 0.05, ** *p* < 0.01, *** *p* < 0.001 (Figure 2d,e). The primer sequences are presented in the attached Table S1.

**Figure 3:**
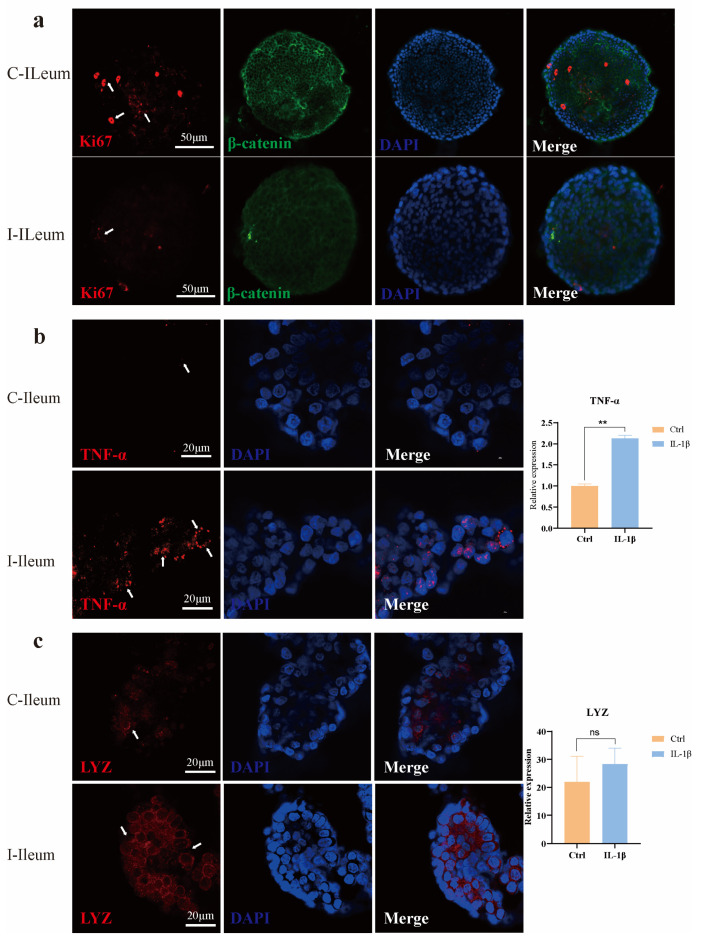
The combined analysis results of immunofluorescence and qPCR of ileum organoids in East Friesian sheep. (**a**) Proliferating cells (Ki67) were co-stained with adhesion junction marker β-catenin via immunofluorescence. Scale bar, 50 μm. (**b**) Immunofluorescence (IF) staining of TNF-α and quantitative analysis of its mRNA expression levels. Scale bar, 20 μm. ** *p* < 0.01. (**c**) Immunofluorescence staining of lysozyme and corresponding mRNA expression analysis. Scale bar, 20 μm. ns, no significant difference.

β-catenin, as a key component of the adherens junction complex, cooperates with E-cadherin and α-catenin to maintain cell-cell adhesion and tissue architecture through the regulation of cell proliferation and intercellular cohesion; β-catenin is a key functional effector molecule downstream of the classical Wnt signaling pathway. It is important in embryonic development, tissue homeostasis, and tumorigenesis. Ki67 (proliferative marker) and β-catenin were detected in the control group and the IL-1β treatment group; notably, the proportion of Ki67 positive cells was significantly reduced in the IL-1β-treated group compared to controls, suggesting IL-1β suppresses cell proliferation in ileal organoids. (Figure 3a). Immunofluorescence staining and quantitative analysis were used to investigate the expression of TNF-α in ileum organoids under different treatment conditions. The results showed that the expression of the proinflammatory cytokine TNF-α in ileum organoids was significantly up-regulated after IL-1β treatment (** *p* < 0.01) (Figure 3b). This cytokine exacerbates inflammation by activating NF-κB signaling and promoting the production of downstream proinflammatory mediators.

Lysozyme (LYZ), an antimicrobial peptide with anti-inflammatory properties, was upregulated at both mRNA and protein levels in response to IL-1β treatment, although there was no significant difference in the relative expression level of LYZ (Figure 3c). The relative expression level of LYZ was higher compared with the Ctrl group. This finding suggests that IL-1β may activate the inflammatory signaling pathways of organoids.

### 3.3. Transcriptional Profiling of Intestinal Organoids Revealed Differential Gene Expression Patterns Between the IL-1β-Treated Group and Control Group

**Figure 4:**
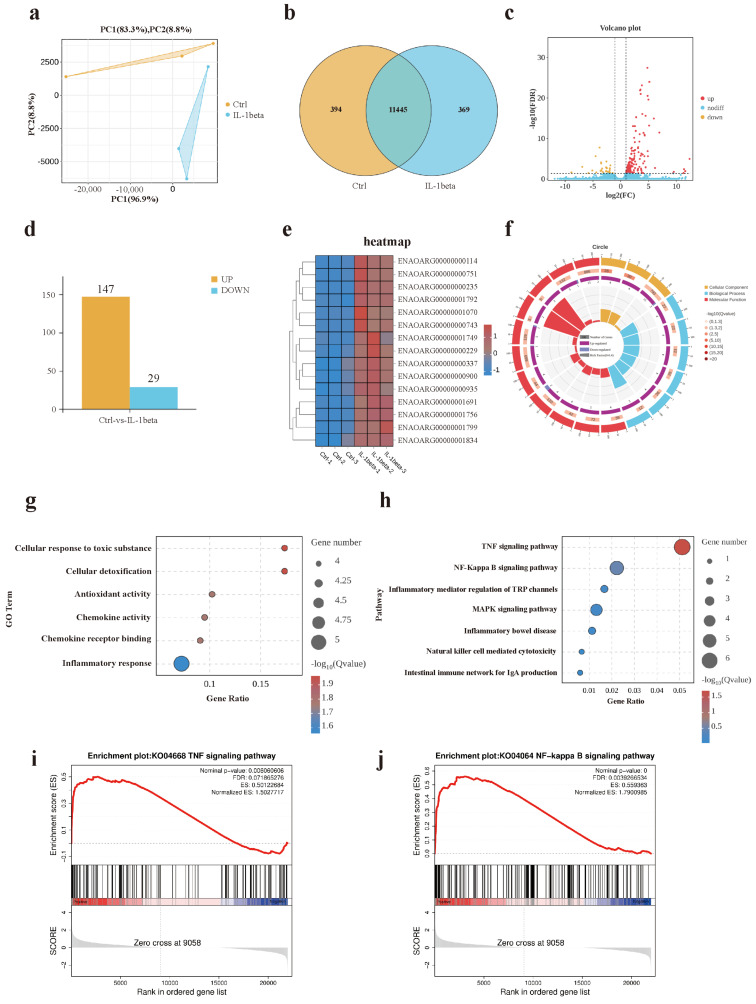
RNA-Seq analysis comparing control and IL-1β-treated groups. (**a**) Principal component analysis (PCA) of global gene expression profiles. (**b**) Venn diagram showing the overlap of differentially expressed genes (DEGs) between groups. (**c**) Volcano plot highlighting DEGs with significant expression changes (adjusted *p*-value < 0.05, |log2 fold change| > 1). (**d**) Bar chart depicting the number of upregulated/downregulated DEGs in each group (n = 3 biologically independent samples). (**e**) Heatmap of DEG expression patterns across samples, clustered by hierarchical clustering. (**f**) Function categories that are significantly enriched under different GO classifications. (**g**) GO enrichment analysis of biological processes (BP), cellular components (CC), and molecular functions (MF). (**h**) KEGG pathway enrichment analysis of DEGs. (**i**) GSEA of TNF-α signaling pathway (NES > 1.5, FDR = 0.07). (**j**) GSEA of NF-κB signaling pathway (NES > 1.5, FDR < 0.05).

Principal component analysis (PCA) revealed a clear separation between the control group and the IL-1β -treated group along the first two principal components (PC1 and PC2), indicating significant differences in global gene expression profiles (Figure 4a). A total of 763 differentially expressed genes (DEGs) were identified, comprising 394 unique genes in the Control group, 369 unique genes in the IL-1β group, and 11,445 commonly expressed genes across both groups (Figure 4b). Among these, 147 genes were up-regulated, and 29 genes were down-regulated in the IL-1β group compared to the Control group (Figure 4c,d). Expression levels of DEGs exhibited marked inter-group variations (Figure 4e). GO enrichment analysis of DEGs highlighted significant enrichment in biological processes such as cellular response to toxic substances, cellular detoxification, antioxidant activity, chemokine activity, chemokine receptor binding, and inflammatory response (Figure 4g). KEGG pathway analysis demonstrated that DEGs were primarily enriched in the TNF signaling pathway (Figure 4h). Furthermore, Gene Set Enrichment Analysis (GSEA) revealed a significant upregulation of both the TNF signaling pathway and NF-ĸB signaling pathway in the IL-1β-treated group compared to the Control group (Figure 4i,j).

### 3.4. NF-κB/TNF Signaling Pathways Were Identified and Uncover Their Roles in Inflammation and Immune Responses

**Figure 5:**
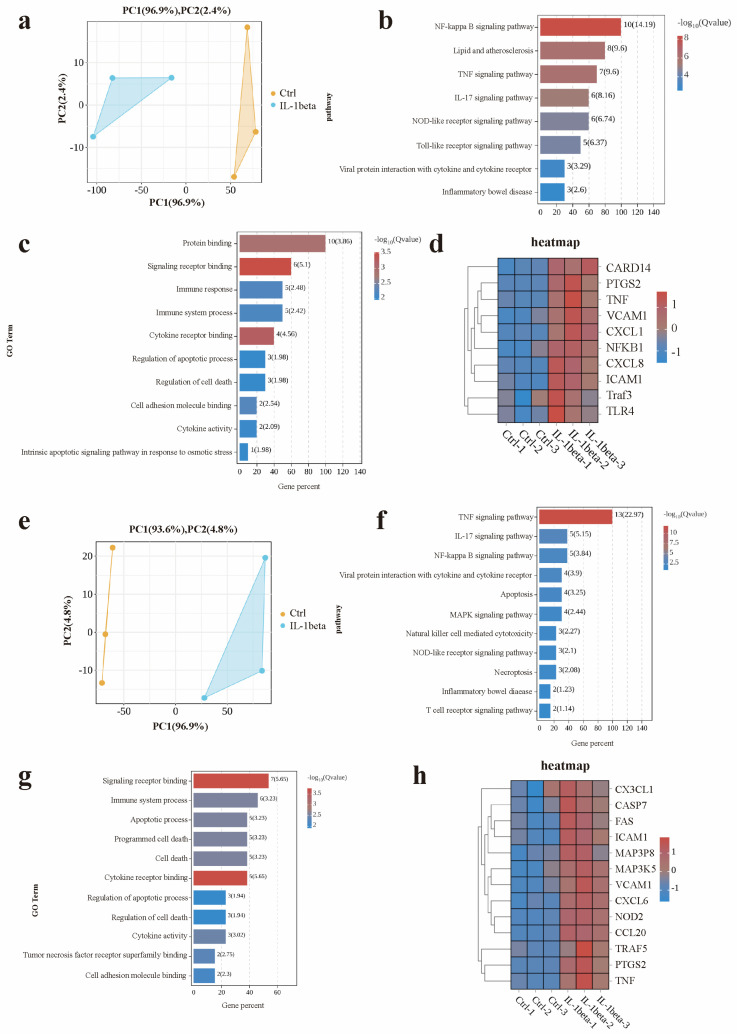
Correlation analysis of the canonical and non-canonical NF-κB signaling pathways. (**a**) Principal component analysis (PCA) of global gene expression profiles in control and IL-1β-treated groups. (**b**) KEGG signaling pathways of canonical NF-κB activation (TNF-α/TLR pathway) and non-canonical NF-κB activation (CD40/RANK pathway). (**c**) GO enrichment analysis of biological processes (BP), cellular components (CC), and molecular functions (MF) for differentially expressed genes (DEGs). (**d**) Heatmap of DEGs associated with canonical NF-κB signaling (e.g., TNF-α, NFKBIA). (**e**) PCA of DEG expression patterns across canonical and non-canonical NF-κB signaling modules. (**f**) KEGG pathway enrichment analysis of DEGs in canonical (e.g., TNF signaling pathway) and non-canonical (e.g., NF-κB signaling pathway) categories. (**g**) GO enrichment analysis of DEGs in canonical vs. non-canonical NF-κB regulatory networks. (**h**) Heatmap of DEGs co-regulated by both canonical and non-canonical NF-κB pathways (e.g., RELA, NFKB1).

Significant genes in the NF-ĸB signaling pathway were analyzed, and principal component analysis (PCA) demonstrated significant separation between the IL-1β -treated and control (Ctrl) groups (Figure 5a). KEGG enrichment analysis revealed enrichment in inflammatory pathways, including the NF-ĸB, TNF, and IL-17 signaling pathways (Figure 5b). GO analysis indicated that these genes were primarily involved in protein binding, signal receptor binding, immune response, and immune system processes (Figure 5c). Gene heat maps showed a marked upregulation of cell adhesion and signal transduction related genes post-IL-1β treatment, including CARD14, VCAM1, and ICAM1. Additionally, inflammation/injury-related genes, such as PTGS2, CXCL1 (a ligand for CXCR2, which was shown to be induced by IL-1β), CXCL8 (a downstream target of IL-1β signaling), Traf3, NF-κB1, TNF, and TLR4 were significantly elevated (Figure 5d). PCA of the TNF signaling pathway genes also showed complete separation between the IL-1β and Ctrl groups (Figure 5e). KEGG analysis confirmed enrichment in the TNF, IL-17, and NF-κB signaling pathways, as well as other inflammatory response-related pathways (Figure 5f). GO enrichment analysis highlighted pathways involved in signal receptor binding, immune system processes, apoptosis, and cell death (Figure 5g). Compared with the Ctrl group, pro-apoptotic genes (CASP7, FAS) and inflammation-related genes (ICAM1, NOD2, CCL20, TRAF5, TNF) were upregulated in the IL-1β group, while the anti-apoptotic gene CX3CL1 was also significantly increased (Figure 5h). Both the NF-κB and TNF signaling pathways were activated in the IL-1β group, with elevated expression of inflammatory factors (NF-κB1, ICAM1, NOD2, CCL20) and damage/antibacterial genes (CXCL6, CXCL8) compared to Ctrl (Figure 5a–f).

## 4. Discussion

Although immobile cell lines provide foundational insights into intestinal epithelial function and serve as initial platforms for drug screening, they inherently lack the three-dimensional architecture and multicellular complexity of the gut. In the context of inflammatory bowel disease (IBD) modeling, two-dimensional monolayer cultures fail to capture the dynamic intercellular interactions and signaling cascades that characterize the inflammatory microenvironment. In contrast, three-dimensional intestinal organoids derived from LGR5+ stem cells can self-organize into structures mimicking primary intestinal tissue, incorporating polarized epithelia, extracellular matrix interactions, and complex multicellular communities [26].

Intestinal organoids can simulate the structure and function of the sheep intestine in vitro. These tiny three-dimensional structures were cultured from LGR5+ stem cells derived from sheep intestinal crypts and were able to reproduce a wide range of cell types in the gut, including absorbent intestinal cells, goblet cells, and Paneth cells, as well as other intestinal endocrine cells [27]. The culture of intestinal organoids begins with single or multiple stem cells, which can be expanded indefinitely under specific culture conditions and can differentiate into all the major cell types of the intestinal epithelium. Over time, the cells self-organize into a spherical or cystlike body with a complete intestinal epithelium containing hollow chambers that mimic the inner cavity of the gut [28].

Our sheep organoid system provides a more controllable and simplified research platform compared to conventional animal inflammation models. While traditional animal models are subject to confounding factors such as microbiome variations, systemic immune responses, and environmental influences, our ovine organoid model enables a focused investigation of specific cellular interactions and the molecular mechanisms underlying inflammation.

Using a novel in vitro model of intestinal organoids, we compared the morphological and functional phenotypes of intestinal organoids in non-inflammatory bowel disease controls with those treated with the inflammatory factor IL-1β. Compared with the control group, organoids treated with IL-1β showed significant morphological and inflammatory mechanism changes, such as shrinking of organ morphology, decreased bud formation ability, and increased secretion of inflammatory factors. Intestinal organoids that grew for 3–4 days after passage were induced by inflammatory factors. The results showed that a certain concentration of inflammatory factors had an impact on the morphology of organoids; the overall area of organoids shrank, and the intermediate cells began to suffer apoptosis. Further application in IL-1β-induced models of intestinal inflammation, the expression levels of NF-κB signaling pathway, TNF signaling pathway, IL-17, and other inflammation-related signaling pathways were seriously up-regulated, including the expression levels of their downstream related factors. The TNF signaling pathway was activated by IL-1β, and real-time PCR proved that the downstream death domain proteins TRADD and FADD of the TNF signaling pathway were activated, which further activated a series of apoptotic proteases such as caspase-3 and finally activated the expression of apoptosis-related factors such as CCL20 and CXCL5. In addition, after the action of IL-1β, the domains of the two intracellular receptors IL1-R and Toll-like receptor (TLR) are close to each other, and IL-1R-related kinase 1 (IRAK1) and tumor necrosis factor receptor-related factor 6 (TRAF6) are activated by recruiting cytoplasmic myeloid differentiation primary reactive protein 88 (MyD88), and finally activate nuclear factor kB (NF-kB), resulting in the increased expression levels of inflammation and immune-related factors such as IL-8, COX2, and NOD2. In addition, there was a significant decrease in the expression of tight junction proteins in ovine intestinal inflammation organoids, revealing impairment of epithelial barrier function in these cell models in the disease state.

**Figure 6:**
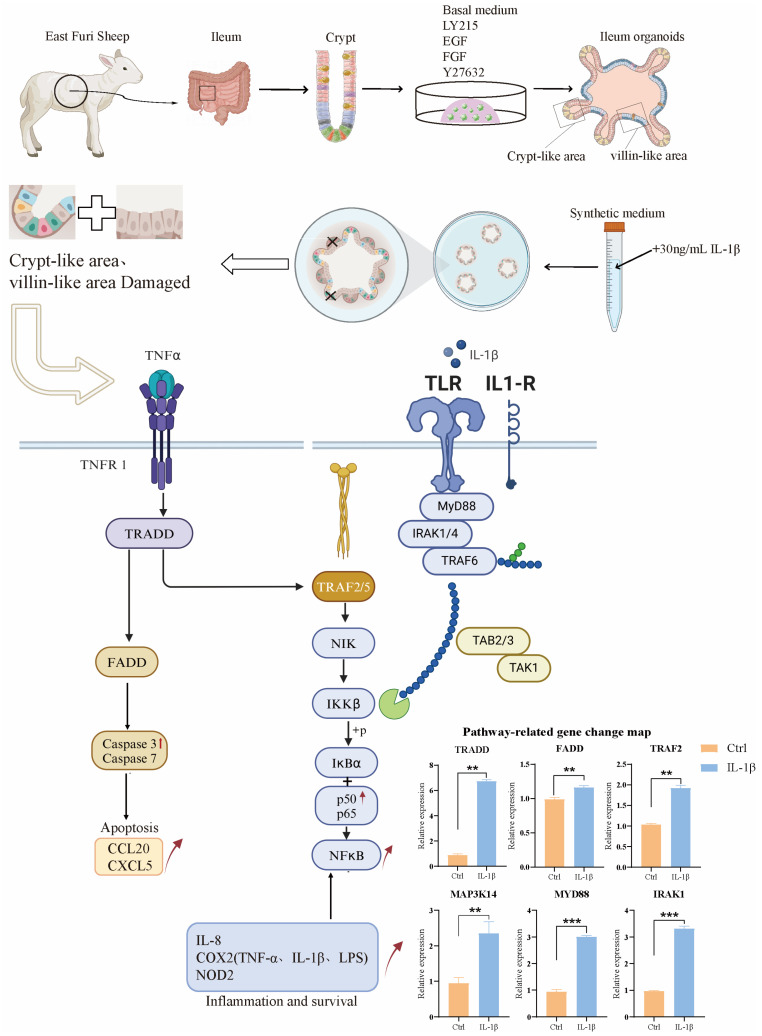
Diagram of the overall experimental pattern. ** *p* < 0.01, *** *p* < 0.001.

These models enable the study of ovine intestinal inflammation pathogenesis, including the dysregulation of tight junction proteins and the upregulation of inflammatory pathways such as NF-κB, TNF, and IL-17 signaling. Specifically, our study established ileal organoid models from East Friesian sheep, demonstrating their capability for continuous passaging, cryopreservation, and disease simulation. Treatment with the proinflammatory cytokine IL-1β induced significant morphological changes, including reduced bud formation and increased apoptosis, alongside elevated secretion of inflammatory mediators. Mechanistically, IL-1β activated the MyD88-TRAF6 signaling axis, leading to the downstream activation of caspase-3 and the proinflammatory cytokines IL-8, CCL20, and CXCL5. These findings underscore the utility of sheep intestinal organoids as physiologically relevant models for translational research (Figure 6).

However, this study also has certain limitations. When it comes to the sheep intestinal organoid model, the establishment and maintenance of sheep intestinal organoids are technically challenging. The isolation of stem cells from sheep intestines requires precise and complex procedures, and the subsequent culture conditions need to be delicately optimized. Small deviations in any step can lead to poor organoid growth or even the failure of establishment.

Secondly, compared with some commonly used model organisms like mice, the cost of obtaining sheep samples and conducting related experiments is relatively high. Sheep farming requires a large amount of space, feed, and labor, which not only increases the economic burden of the research but also limits the scale of sample collection and experimentation.

In addition, although sheep intestinal organoids can mimic some aspects of the in-vivo intestinal environment, they still cannot fully replicate the complex physiological and pathological conditions of the entire sheep body. For example, the interaction between the intestine and other organs in the body, as well as the influence of the systemic immune system on the intestine, may not be accurately reflected in the organoid model. There may also be differences in the response of sheep intestinal organoids to certain stimuli compared to the real-time in-vivo situation.

Finally, the existing knowledge and research experience on sheep intestinal organoids is relatively limited compared to well-studied model systems. This lack of in-depth understanding may pose difficulties in data interpretation and further in-depth exploration of the model, restricting the wide application and development of this model in scientific research.

## 5. Conclusions

In this study, We managed to create an in vitro organoid model from the ileal crypt of East Friesian sheep. We proved these gut organoids can mimic the sheep’s gut structure and function. Then, we used the inflammatory factor IL-1β to build an intestine inflammation disease model. The treated organoids showed major changes in shape and inflammation compared to the control group. RNA sequencing revealed that IL-1β triggers gut inflammation by promoting the NF-Kappa B/TNF/IL-17 pathway. Further investigation was conducted on the pathogenesis of IL-1β in Intestinal inflammation.

## Data Availability

The datasets used and/or analyzed during the current study are available from the corresponding author upon reasonable request due to ethical and legal restrictions regarding participant privacy.

## Supplementary Materials

The following supporting information can be downloaded at https://www.mdpi.com/article/10.3390/ani15081097/s1: Figure S1: IL-1β concentration screening map; Table S1: RT-qPCR Primers Sequences.
