# Supplementary material for: IL-1β Induced Intestinal Inflammation Pathogenesis in East Friesian Sheep: Insights from Organoid Modeling
Source: Animals (Basel). 2025 Apr 10;15(8):1097. doi: 10.3390/ani15081097 (PMC12024061; doi:10.3390/ani15081097)
Supplement: Supplementary file 1 [file animals-15-01097-s001.zip › Table S1.docx]

**Supplementary Tables**

Table S1. RT-qPCR Primers Sequences

| Name | Sequence (5’ to 3’) |
| --- | --- |
| 18SRNA-forward | GTAACCCGTTGAACCCCATT |
| 18SRNA-reverse | CCATCCAATCGGTAGTAGCG |
| OLFM4-F | TTCTCCTAGCCCTTCTGTTCTTC |
| OLFM4-R | TTCCAAGCGTTCCACTCTGTCC |
| Ki67-F | TCCTTTGGTGGGCACCTAAGACCTG |
| Ki67-R | TGATGGTTGAGGTCGTTCCTTGATG |
| MUC2-F | GCCAAGAATCCGGGACATCT |
| MUC2-R | CACATCCACCAGCTCTCGTT |
| CHGA-F | GGCTACCCAGAGGAGAAGAAGGA |
| CHGA-R | CTCCAGCTGGTTGGACACCTT |
| LGR5-F | TTCACTTTCGGCAGCTTTGC |
| LGR5-R | TGGGAAAGGGAGTTTGCGTT |
| LYZ-F | CGCTGTCCAAGCCAAGGTC |
| LYZ-R | TGCCAGACTGATTCCCCTAA |
| DCLK1-F | ATAGCTTCATCGTCCCGCAG |
| DCLK1-R | CGTGAAACCTTCACCCAACC |
| VILL1-F | GGACCTGTGGTACCGTGAAG |
| VILL1-R | AAGTTGACGATCTCGGCTGT |
| TRADD-F | CGGCCAGGAGCAAGATGG |
| TRADD-R | CTGGTGAGCTCGTTCTCCTC |
| FADD-F | ACCTGACCCAGCTCAAGTTC |
| FADD-R | CGGGGGTACTTCTCCTCGAT |
| TRAF2-F | GGACAAGTACCTGTGCTCGG |
| TRAF2-R | TTCGTGGCAGCTCTCGTAGT |
| MAP3K14-F | TGTGCCGTCAAAAAGGTTCG |
| MAP3K14-R | AACAGCTGCTCCAGATGTCC |
| Caspase3-F | ATGGGAGCAAGTCAGTGGAC |
| Caspase3-R | AGCGAGATGACATTCCAGTGC |
| MYD88-F | CTCATTGAGAAGAGGTGCCG |
| MYD88R | GGTGCAGGGGTTAGTGTAGTC |
| IRAK1-F | TCCCCGCAAGAGAACTCCTA |
| IRAK1-R | CAGGAGGGGTCCTTCCACA |
