# Supplementary figures and images for: IL-1β Induced Intestinal Inflammation Pathogenesis in East Friesian Sheep: Insights from Organoid Modeling
Source: Animals (Basel). 2025 Apr 10;15(8):1097. doi: 10.3390/ani15081097 (PMC12024061; doi:10.3390/ani15081097)

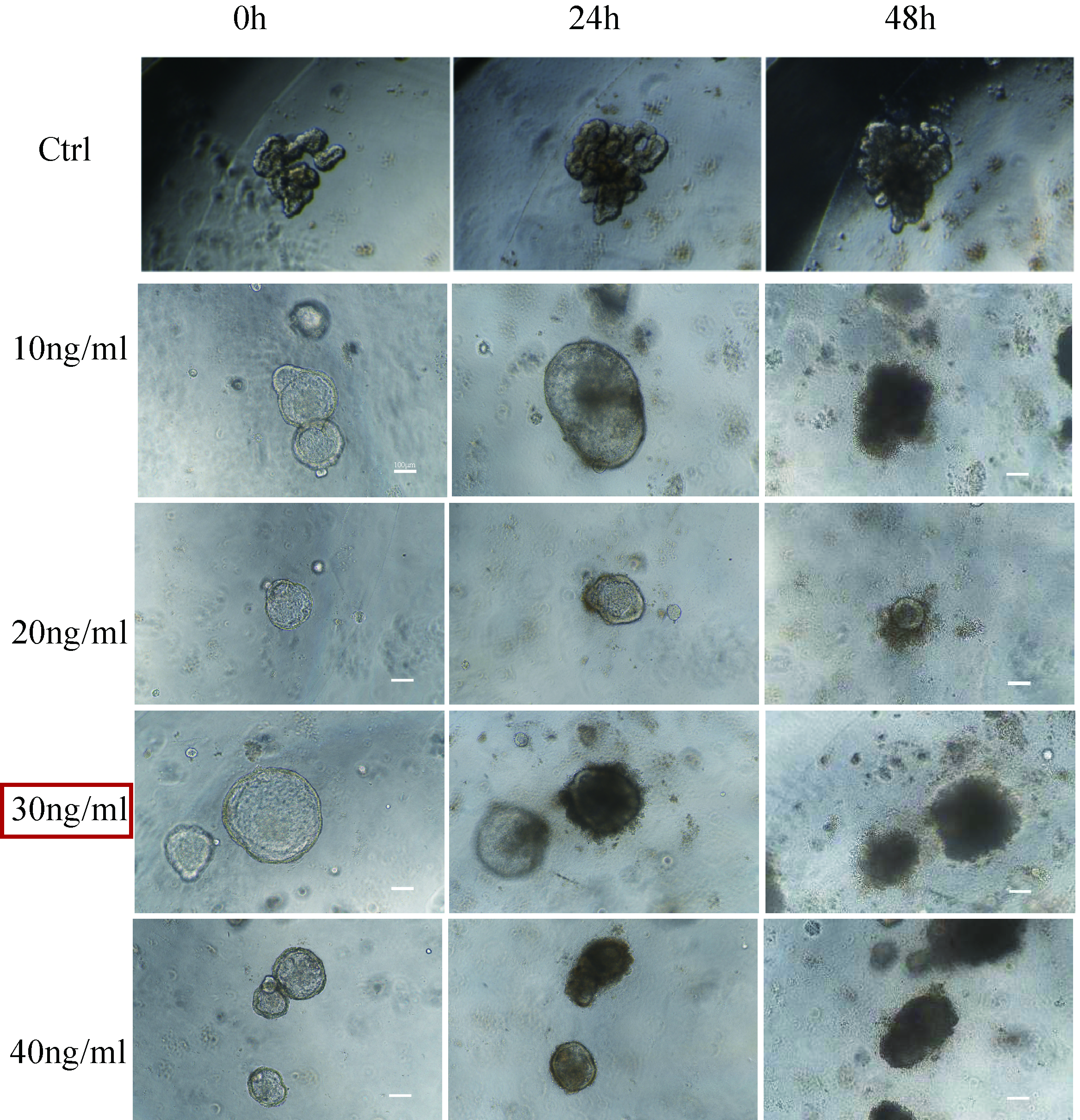

Supplement: Supplementary file 1 [file animals-15-01097-s001.zip › Figure S1.tif]
